# Unraveling Endothelial Cell Migration: Insights into Fundamental Forces, Inflammation, Biomaterial Applications, and Tissue Regeneration Strategies

**DOI:** 10.1021/acsabm.3c01227

**Published:** 2024-03-23

**Authors:** Dominika Jerka, Klaudia Bonowicz, Klaudia Piekarska, Seyda Gokyer, Utku Serhat Derici, Osama Ali Hindy, Baris Burak Altunay, Işıl Yazgan, Kerstin Steinbrink, Konrad Kleszczyński, Pinar Yilgor, Maciej Gagat

**Affiliations:** † Department of Histology and Embryology, Collegium Medicum in Bydgoszcz, Nicolaus Copernicus University in Torun, 85-092 Bydgoszcz, Poland; ‡ Faculty of Medicine, Collegium Medicum, Mazovian Academy in Płock, 09-402 Płock, Poland; § Department of Dermatology, University of Münster, Von-Esmarch-Str. 58, 48149 Münster, Germany; ∥ Department of Biomedical Engineering, Faculty of Engineering, Ankara University, Ankara 06100, Turkey

**Keywords:** cell migration, endothelial cells, biomaterials, inflammation, angiogenesis

## Abstract

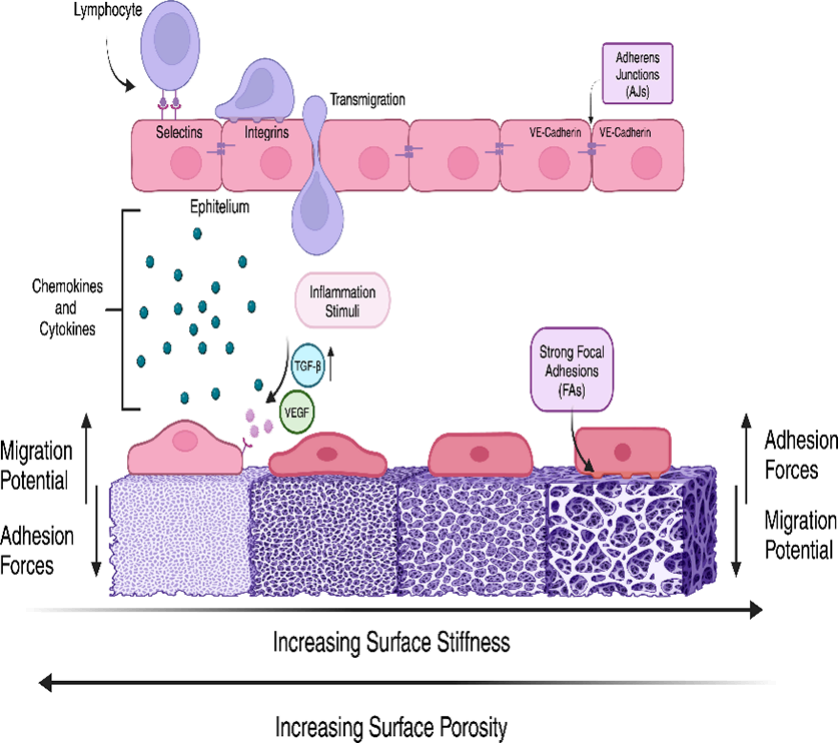

Cell migration is vital for many fundamental biological processes and human pathologies throughout our life. Dynamic molecular changes in the tissue microenvironment determine modifications of cell movement, which can be reflected either individually or collectively. Endothelial cell (EC) migratory adaptation occurs during several events and phenomena, such as endothelial injury, vasculogenesis, and angiogenesis, under both normal and highly inflammatory conditions. Several advantageous processes can be supported by biomaterials. Endothelial cells are used in combination with various types of biomaterials to design scaffolds promoting the formation of mature blood vessels within tissue engineered structures. Appropriate selection, in terms of scaffolding properties, can promote desirable cell behavior to varying degrees. An increasing amount of research could lead to the creation of the perfect biomaterial for regenerative medicine applications. In this review, we summarize the state of knowledge regarding the possible systems by which inflammation may influence endothelial cell migration. We also describe the fundamental forces governing cell motility with a specific focus on ECs. Additionally, we discuss the biomaterials used for EC culture, which serve to enhance the proliferative, proangiogenic, and promigratory potential of cells. Moreover, we introduce the mechanisms of cell movement and highlight the significance of understanding these mechanisms in the context of designing scaffolds that promote tissue regeneration.

## 1 Introduction

Cell migration is vital for many fundamental biological processes and human pathologies throughout our life. Although the multistep process of cellular movement follows a specific pattern, the cell type and the context of movement can influence the detailed mode of migration.^1,2^ Cells can move independently using single-cell migration. Otherwise, they can be linked together to move as a cohesive sheet of cells during collective cell migration.^3^ Broadly speaking, cell migration is a dynamic process that consists of four stages. First, the cell’s polarization with regard to external stimulation appears. Soon afterward development of a protrusion at the front edge begins, which leads cells to attach to neighboring cells or the extracellular matrix (ECM), and eventually, the retraction of the trailing edge that propels the cell forward occurs. Disruptions that occur at any of these stages can have significant after-effects.^4^

The endothelium serves as a barrier between the blood flowing inside the vessel and the adjacent tissues.^5^ In fact, endothelial cells can selectively infiltrate immune cells and produce various factors, including vasoactive substances and cytokines. The proper functioning of this single layer of cells is reflected in the efficient functioning of the entire circulatory system.^6^ Vascular endothelial cells (ECs) have recently been classified as a type of innate immune cell due to the gatekeeping of immune responses.^7,8^ ECs are constantly exposed to noxious stimuli and conditions, such as infection and tissue injury, which trigger inflammation as an adaptive response and serve the purpose of returning the damaged endothelium to a healthy state.^9^ However, the activation of inflammatory cytokines and signaling regulators after exposure to infection or various stresses that exceed the capacity of the cells to react plays a key role in the pathological function of normal cells and cancer cells in the tissue microenvironment.^10^ ECs migrate during neovascularization, including vasculogenesis and angiogenesis, but also in a damaged vessel to restore vessel integrity.^1,11^ In normal vascular homeostasis, the formation of new vessels is stimulated by a number of proangiogenic factors, including vascular endothelial growth factor (VEGF).^12^ However, in chronic inflammation, the same factor may induce excessive and pathological angiogenesis, which is also associated with a change in the properties of cytokines in the inflammatory microenvironment.^13^

Biocompatible and biodegradable biomaterials are perfect solutions for regenerative medicine. Materials with such properties serve as tools for tissue substitution, regeneration platforms for injured tissues, and vehicles for drug delivery. Selection of biomaterial is made with regard to its features, such as stiffness, porosity, and surface characteristics, that elicit microenvironmental signals that alter cellular behavior. Previously, the validity of the immunological impact of biomaterial implantation was disregarded. Nowadays, it is increasingly recognized as a critical aspect for effective tissue regeneration. Certain biomaterials exhibit excellent hemostatic properties, including blood-clotting ability, erythrocyte and platelet aggregation, and notable hemocompatibility. These characteristics are pivotal for advancing therapeutic strategies in wound healing and tissue repair. An increased knowledge of healing mechanisms and biomaterial properties can affect the modulation of immune responses. Therefore, it is fundamental for innovative biomaterial designs.^14−19^

Here, we provide a summary of current knowledge concerning how inflammation can potentially influence endothelial cell migration. Additionally, we delve into the fundamental forces that govern cell motility with a specific focus on ECs. Furthermore, we discuss the application of biomaterials in EC cultures, which enhances the proliferative, proangiogenic, and promigratory potential of cells. Lastly, we introduce the mechanisms of cell movement and underscore the importance of comprehending these mechanisms when designing scaffolds to promote the regeneration of injured tissues.

## 2 Cell Migration

Cell migration is a fundamental element determining a variety of processes.^20^ It plays a vital role in ensuring proper immune responses, tissue repair, and homeostasis.^1,21^ In the majority of the migration modes, the success of this course is mostly mediated by Rho-GTPase protein activity. However, there are other factors, such as Cdc42, that control cell polymerization and filopodia formation. Furthermore, lamellipodia, which constitute other membrane protrusions, are dependent on the Rac1 activity. Owing to RhoA-dependent focal adhesion (FA), cells are capable of gripping the ECM. Actomyosin contraction pulls the whole cell forward through Rho-associated kinase (ROCK)-mediated phosphorylation of myosin light chain kinase (MLCK). Moreover, RhoA activity affects the disassembly of FAs and consistently leads to tail retraction.^22^ Failure to execute this process correctly can result in consistently disastrous outcomes. Inadequate or misdirected cell migration may result in unforeseen deviations, such as chronic wounds that do not heal.^23^ Based on distinct cell morphologies, cytoskeletal dynamics, or adhesive properties, several migration modes can be identified. Some cells utilize a single movement pattern, while others rely on collective migration. The movement itself can be directed toward a chemotactic stimulus or may be entirely random. Despite noticeable differences among migration modes, there are also some recurring factors. Certain features are repetitive and follow a cycle of motility.^1,24^ All types of migration require appropriate factors such as cytoskeletal (re)organization or cell polarization. Cohesion and coupling between cells are mediated by adherens junction proteins that are linked to the cytoskeleton. These components’ fundamental functions influence the cells by generating forces, responding to mechanical or chemical signals, and facilitating cell adherence.^25^

### 2.1 Single-Cell Migration

Individual cell movement is undertaken solitarily, which means that the individuals do not have an impact on each other.^26^ During this mode, many processes take place and depending on their course can affect cell shape changes including cyclic extension, adhesion, and also retraction. Certain types of cells are able to switch from single to collective migration mode.^27^ Single migration based cells must initially detect the physical characteristics of the ECM, and then apply the right forces to move. The cell’s adhesion to its matrix impacts its migratory mode. In turn, adhesion is dependent on the architecture, porosity, composition, and mechanical properties of the ECM.^3,28^ Ameboid and mesenchymal migrations constitute two variations of single-cell movement. The Arp2/3 complex is present in both cases, and it manages to form dendritic arrays of actin filaments at the cell edges. Taking aim on actomyosin contractile arrays and also regarding the phenotype of migration, mesenchymal and ameboid cells are the opposite of each other.

Mesenchymal behavior mainly resembles the general type of movement; therefore, it depends on several major subcellular activities. Initially, the process starts with the formation of membrane protrusions in the cell’s leading edge, which allow a cell to form new connections with its environment. Subsequently, it comes to actomyosin contraction, whereby tension is induced, as well as detachment of contact points at the cell’s trailing edge. It all comes down to enable dragging the cell body with regard to the leading edge.^29^ However, cell polarization is quite weak, and protrusions, such as lamellipodia and filopodia, seem to compete with each other. Strong integrin-mediated adherence to the ECM is another trait that inhibits the effectiveness of mesenchymal motility. Regarding motor activity, myosin is the most significant factor. During mesenchymal-based motion, myosin II is correlated to bundled actin stress fibers. Myosin IIB is present mostly in cell retraction areas, whereas myosin IIA is prevalent all over the cell. Contractile actomyosin stress fibers are essential for the high substrate adherence found in mesenchymal cells. The actin bundles play a critical role in the regulation of membrane protrusion and broadly speaking promote cell migration.

Ameboid mode relies on fast migration velocity, which is also a feature of high polarization that permits these cells to protrude effectively via pseudopods and blebs. This type of movement is mostly used by cells migrating via the ECM, routes, and pores. Ameboid cells crawl owing to pseudopodia, and the migration itself refers to the movement of cells that falls short of stress fibers along with FAs. Despite the lack of strong adhesion, mobility is allowed, which is a key feature of this mode. Unlike mesenchymal cells, myosin II is assumed to generate a squeezing force in amoeboid cells, since it is largely localized to the back of the cells in a structure known as the uropod. Regarding the morphology, these cells are much rounder, which is the result of constant changes in shape through quick extension and retraction of membrane protrusions.^3,23,24,30−32^ This mode enables the cells to adapt to prevailing conditions and also allows the activation of distinct molecular mechanisms between well-defined migration strategies that include movement through a diversity of organs. Furthermore, ameboid cells retain a significant capability for recirculation between the lymphatic as well as blood systems. Ameboid movement can be divided into some particular modes. One of them is based on actin polymerization, while the other one includes blebbing-based motility.^33^

Relating to the broad classification of migration modes, blebby migration is also a successful exemplar of single behavior. However, this type of movement differs significantly from the additional motility mechanisms and involves the generation of hydrostatic pressure, which drives expansion of the blebs. In the course of migration, the front of the cell is responsible for generating a sequence of blister-like protrusions in the moving direction. Blebs are actually referred to as cellular protrusions, which play a crucial role in both development and disease. The mechanics of bleb formation consist of 3 phases: initiation, growth, and retraction. The bleb initially appears to be devoid of filamentous actin. Increased hydrostatic pressure leads to focal rupture of the cortical actin cytoskeleton from the membrane, and consequently, by pushing the membrane outward through the cytoplasmic fluid, it leads to the formation of the blebs. Immediately after this step, actin polymerizes on the bleb membrane to enable the cortex to set up. During growth, the actin cortex begins to accumulate on the plasma membrane of the bleb, owing to which retraction of the bleb is enabled. Some cell types only make use of the blebs to cause movement, while others are able to switch between migration modes depending on the environment, so that migration can be optimized as much as possible.

Pseudopodial motility is distinguished by the fact that the cells can form two crucial types of actin-based protrusive organelles, lamellipodia and filopodia. During this mode, actin is constantly cycled to the cell’s front, which consequently pushes the membrane forward in the moving direction.^34−36^ The lamellipodium is defined as a network of short and branched actin filaments that are directed by the Arp2/3 activated complex. After activation, filaments begin to elongate and barbed-ends are being capped. Filopodia, on the other hand, are transitory, thin, hairlike protrusions with actin bundles. They are thought to be produced by remodeling of the dendritic network.^37^ Their development is influenced by a variety of circumstances, such as actin bundlers, elongators, and Rho-GTPases. Therefore, filopodia are capable of probing local environmental cues. They are in charge of controlling the direction of movement, moreover they support persistence of migratory cells via increasing cell–matrix adhesion at the leading edge.^38^

### 2.2 Collective Cell Migration

Collective migration is described as the movement of individual cells that impact each other’s behavior and are capable of forming groups that are strongly or loosely linked. Unlike single-cell behavior, collectively migrating cells migrate much more quickly and more precisely. Thus, the primary feature of this mode is more efficient cell movement. Despite the fact that single cells have a higher instant velocity, they migrate in a less durable manner, changing direction frequently. Such collective behavior requires physical or chemical crosstalk among individuals. However, cells that are characterized by collective behavior can respond to mechanical signals in the same way as single cells. It all comes down to the fact that the cells must assimilate information from their surroundings. Furthermore, collective-based cells coordinate their response to the environment, making sure that immobile cells or those that migrate in a different direction also go along with the global flow. Based on widely reported cell migration studies, it was confirmed that the fundamental mechanics of single-cell migration may be applied to collective movement, as well. During group migration, the cells stay attached throughout the process, allowing cell–cell junctions to remain intact during movement.^3,26,28,38−40^ Collective migratory behaviors occur in response to a variety of environmental limitations, which result in a range of morphological characteristics. They include the presence of mesenchymal leader cells that are highly motile, cryptic lamellipodia development by cells placed a few rows behind the leading front and the formation of small groups of follower cells that are directed by the leader ones.^41^ This type of cell migration relies on front–rear polarity. Cells collected ahead become the leaders in regard to external cues and enlarge lamellipodia or filopodia on the road to the substrate. When these pressures are applied, the matrix physically deforms, forming a route for the whole cohort.

By and large, mesenchymal behavior allows the immobile cells to move through the mobile ones that carry them. Migrating cells interact with each other, which consequently provides the relevant cell distribution.^23,42^ To enable cell–cell interaction, adhesive proteins such as cadherins are required. Owing to these, contact inhibition of locomotion (CIL) occurs. CIL is referred to as a process during which a cell ceases to move once it is touched by another cell. This mechanism consists of two phases. The first one takes place during contact and involves cell protrusions collapsing, which leads to a brief halt in migration, whereas the second stage includes repolarization in the reverse direction, where cells progressively move away from one another. CIL suppresses the development of cell protrusions between neighbor cells. As a result, the majority of protrusive activity is directed toward the open space.^43^ Despite the fact that mesenchymal cells are characterized by unstable cell–cell junctions, migration as a loosely linked pack is achievable and the repulsion between cells occurs as a result of cell–cell contact.^26,44^

**Figure 1:**
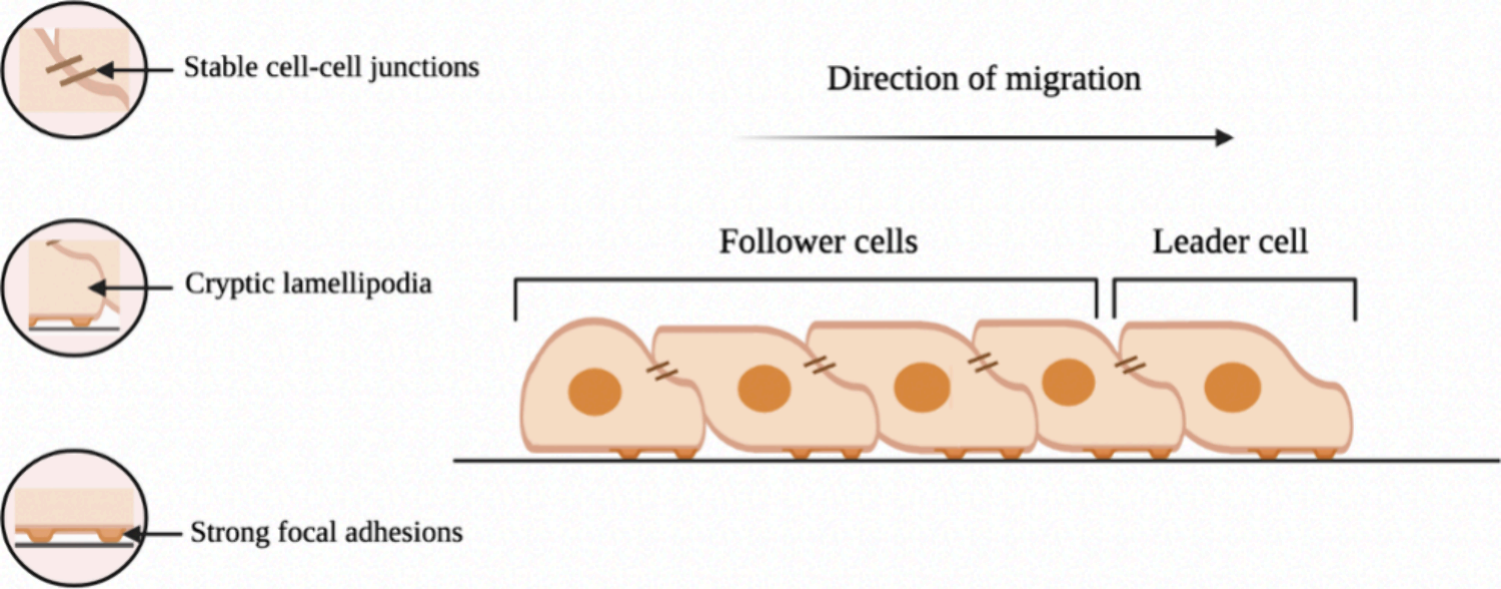
Collective migration of epithelial cells. Leader cells begin migration, whereas follower cells trail behind them. Cadherin stable junctions connect cells and allow them to migrate with the lamellipodia. Owing to strong focal adhesions, each cell makes an equal contribution in migration (designed with BioRender: https://biorender.com/).

Epithelial cells, as well as endothelial cells, are two types of cells that are responsible for lining body surfaces. Therefore, endothelial cells are a specific kind of epithelial cells. The primary distinction between these types of cells is that epithelial cells line both internal and exterior surfaces of the body, whereas endothelial cells line the interior surfaces of circulatory system components.^45^ Epithelial migration is essential for development, morphogenesis, tissue homeostasis, and wound healing. It is assumed that epithelial cells move by exerting locomotor pressure on the ECM. However, they maintain physical contacts with one another to maintain tissue integrity. During migration, epithelial cells retain persistent cell–cell junctions. In the course of time, when two epithelial cells come into touch, they probe each other using Rac1-driven lamellipodia. Owing to these protrusions, cadherins from two neighboring cells are allowed to connect to form adherens junctions (AJs). The lamellipodia immediately collapse at their primary contact point and advance sideways to expand the region of the interaction. A crucial function is also performed by small GTPases, whose activity maintains contractility at intermediate levels. Epithelia are quiescent, and the cells are “jammed” in their specified positions. Migration of epithelial cell groups, such as those taking part in wound healing, is allowed by tissue fluidization through an unjamming transition in which tension is decreased. During such events, the cell adhesion is considered high and every cell contributes equally to the movement of the group. This type of collective migration is characterized by the fact that leader and follower cells produce both traction forces that pull on the substrate and then protrusions that are oriented toward the direction of migration. Unlike mesenchymal cells, in epithelia strong intercellular connections hold cells together and repulsion does not take place (Figure 1).^26,44,46^

## 3 Endothelial Cell Migration in Normal and Proinflammatory Microenvironments

Endothelial homeostasis is vital for proper cardiovascular function and therefore is required for tissue homeostasis during the lifetime of an organism.^47^ It is conceptualized as a successful balance between endothelial injury and regeneration, enabling the proper response to noxious chemical and mechanical signals. An inseparable and fundamental element of the growth and repair of the normal and diseased vessel wall is the process of cell migration.^48^ The endothelium is a monolayer of endothelial cells in direct contact with the flowing blood, constantly exposed to different stimuli, which may either inhibit or enhance EC actions.^49^ In the circulatory system, shear stress has a critical role in initiating the signaling cascade, leading to EC activation and dysfunction. Consequently, accumulating cytokines and growth factors may affect the migration of endothelial cells, which is often parallel with blocked or inappropriate endothelial regrowth after injury, an underlying cause of vascular pathology.^50^ Besides restoration of vessel integrity, a crucial phenomenon based on cell migration and driven by endothelial cells is the formation of new blood vessels, observed under both physiological and pathological conditions. Blood vessel formation occurs through vasculogenesis and angiogenesis.^51^ There are significant differences in the course of these processes, depending on the microenvironmental conditions. Vasculogenesis is described as the *de novo* formation of blood vessels from progenitor cells. Consequences of disrupted vasculogenesis can be seen in a variety of congenital disease states. Angiogenesis is the formation of new vessels from existing ones, such as capillaries, a process often contributing to inflammatory diseases or states in an adult organism and therefore relevant to this review.^52^ Although any human system or tissue can be affected by the inflammatory process, which is always mediated by endothelial cells, its molecular basis and cellular mechanisms are remarkably consistent, regardless of where it occurs.

### 3.1 Vascular Repair via Endothelial Migration

The maintenance of vascular homeostasis is an active process that, apart from migration, is involved in most cellular processes, such as growth, activation of immune cells, and production and degradation of the ECM, which must coordinate with microenvironmental stimuli to maintain blood vessel function.^53^ The physiological role of the endothelium is based on its ability to produce cytokines and adhesion molecules that regulate and direct inflammatory and regenerative processes. Therefore, the endothelium modulates the structure and physicomechanical properties of the vascular walls over time and profoundly affects vascular health. EC migration occurs according to the leader–follower model, involving coordinated processes of chemotaxis, haptotaxis, and mechanotaxis, which requires fine-tuning of EC migration.^54,55^ Endogenous and exogenous reparative mechanisms serve to restore a functional endothelial monolayer and re-establish the disrupted endothelial cell–cell junctions to reform a semipermeable barrier. The stability of endothelial cell contacts is ensured by AJs, tight junctions (TJs), and gap junctions (GJs). AJs and TJs form molecular connections between adjacent ECs, promoting a strong bond along the EC perimeters. In contrast, gap junctions do not contribute to homotypic attachment but instead form intracellular channels, allowing for chemical and electrical communication between neighboring ECs.^11,56^ Collective migration is defined as the migration of cells that remain functionally and physically connected through stable intercellular connections. Endothelial regeneration may involve resident ECs themselves (i.e., endogenous) or cells other than the resident EC population, such as circulating stem/progenitor cells (i.e., exogenous). In that event, the behavior of directed collective migration throughout the sheets is observed. Moreover, this dynamic planar migration maintains the integrity of the monolayer in a process that likely involves dynamic cadherin turnover at the cell junctions. Recovery of damaged blood vessels, not correlated with disease risk, usually requires only a combination of resident EC elongation and migration from both ends of the lesion site and resident EC proliferation.^57^

**Figure 2:**
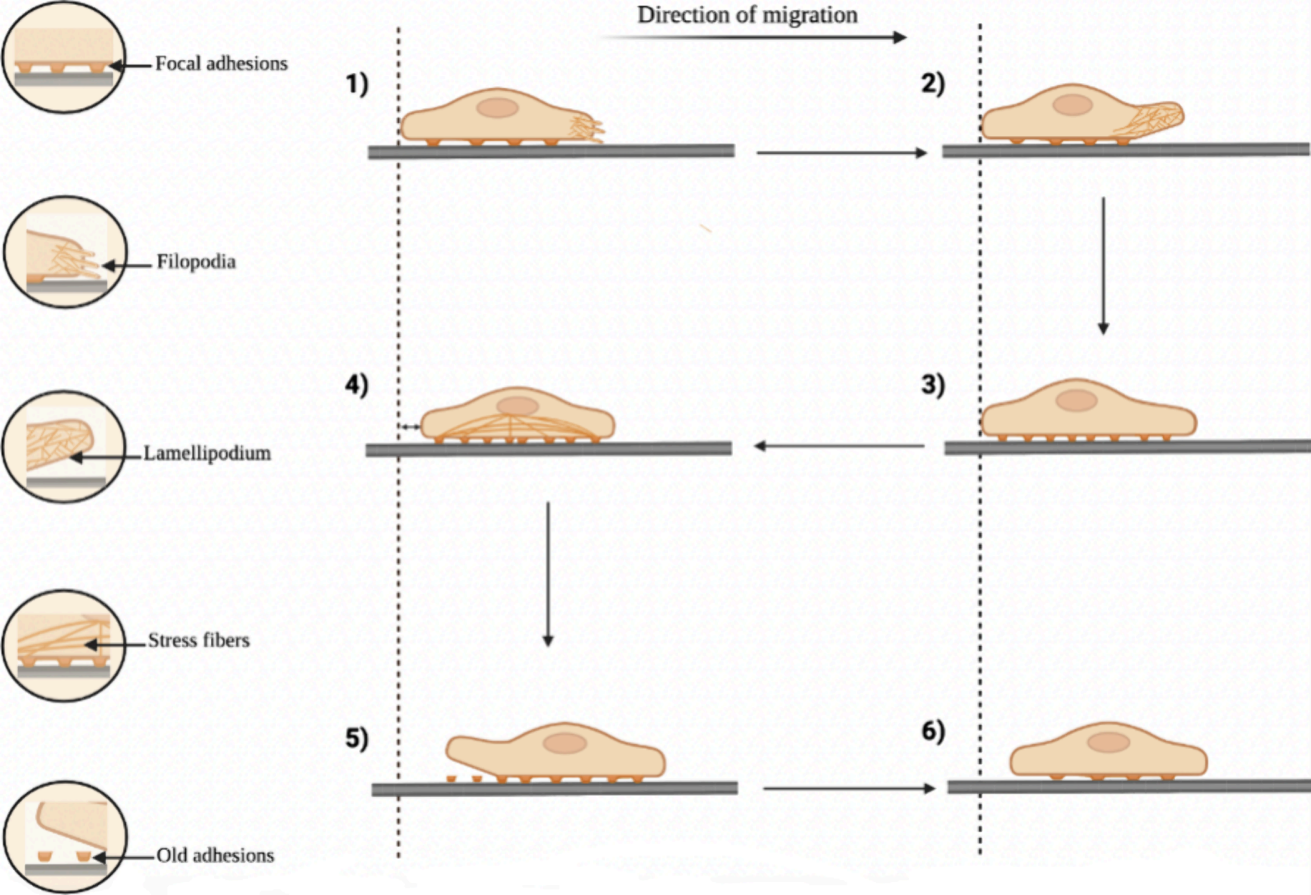
Six crucial steps of endothelial cell migration. (1) Filopodia detection of motile stimuli. (2) Cellular extension of protruding lamellipodium. (3) Attachment of the protrusions to the ECM. (4) Stress fiber-mediated contraction and translocation of the cell. (5) Rear release of the cell edge. (6) Recycling of membrane receptors from the cell’s rear to its front (designed with BioRender: https://biorender.com/).

Filling the gap in the endothelium by migrating cells is a locally coordinated process promoted by growth factors, such as the basic fibroblast growth factor (bFGF). Cells triggered by growth factors near the wound edge exhibit collective migration of the sheet toward the denuded area. More specifically, enhanced growth factor signaling results in the steering of boundary cells that provides a directional cue given by the location where AJs are absent. The directed motility in boundary/pioneer cells is sufficient to generate movement of an entire interconnected monolayer, biasing the movements of follower and neighboring cells in the forward direction. In migration not stimulated by any growth factor, the cell movement is more random and stops when the injury area is covered by cells.^1,58^ The regulation of single-cell motility within the monolayer is inseparably dependent on actin filament polymerization, which determines the direction and the speed of movement and membrane protrusion at the fronts of cells.^59^ The actin-based membrane protrusions must also generate traction forces through contact with the ECM, exhibiting high and critical pseudopodial activity.^60^ Cells that migrate collectively are characterized by multicellular polarity and the “supracellular” organization of the actin cytoskeleton. In nonactivated endothelium, F-actin is organized into characteristic star-like structures that guarantee an appropriate distribution of intracellular tensions and enable a quick response to external factors, for example, through the ability of multidirectional migration (Figure 2).^61^

Vascular inflammation involves a complex network of interactions that begin as a beneficial repair process; however, the same inflammatory processes play an important role in the initiation and development of chronic diseases such as atherosclerosis and cancer in all stages.^62^ Acute vasculitis begins due to the imbalance between vasodilation and vasoconstriction factors in the endothelium, which is greatly mediated by endothelial nitric oxide (NO) level and contributes to endothelial dysfunction.^63^ Impaired bioavailability of endothelial nitric oxide synthase (eNOS)-produced NO is also associated with vascular hyperpermeability and infiltration of inflammatory mediators and immune system cells.^64,65^ Simultaneously, changes occur at the membrane protein level, manifested by unsealing of intercellular junctions.^66^ Prolonged inflammation is, next to mechanical stimuli, the principal cause of endothelial damage, mainly caused by the excess of inflammatory cytokines and accumulating immune cells including T cells and monocytes. Endothelial cells are capable of expressing a range of cytokines and transcription factors that promote proliferation, migration, and endothelial healing, whereas some of these inflammation-related molecules are related to dysregulation of vascular repair *in vitro* and *in vivo*, and the establishment of chronic inflammation.^10,67^ Recent scientific reports investigated the relationship between vascular stiffness and monolayer healing following a vascular injury. Disease states related to inflammation, such as diabetes or atherosclerosis, are characterized by stiff vessels and may contribute to poor vascular healing. It has been shown that stiffer substrates enable a farther EC collective migration, while the softest substrates have the highest degree of intact cell–cell junctions. In addition, adequate substrate stiffness is required to maintain a balance of actomyosin contractility and affects the sensitivity to exogenous signals. This bidirectional dependence also explains that EC interactions with the ECM and cell–cell adhesion structures are required to adjust their mechanical properties and exert the appropriate forces enabling the movement of cells in response to transduced biochemical stimuli. The latest findings also show that subendothelial stiffness, beyond the critical change of endothelial cells’ mechanical behavior, can also affect their transcriptome as evidenced by upregulated expression of TGF-β2 on stiffer matrices and different responses to exogenous cytokines including TGF-β2.^28,59,68^ M1 macrophages are formed in a proinflammatory environment under the influence of lipopolysaccharide and proinflammatory cytokines, such as TNF-α and IFN-γ. These immune cells have been shown to secrete chemokines associated with the acute inflammatory phase, such as IL-1β, MCP-1, and MIP-1α; however, analysis of their secretome also revealed the presence of proangiogenic factors. The existence of M1 macrophages induces cell detachment and strong single-cell migration, suggesting that vascular cells migrate in an inflammatory environment without immediately forming blood vessels. It was also observed that chronic wounds, characterized by prolonged inflammation, exhibit inadequate angiogenesis leading to impaired wound healing. Thus, it is likely that the onset of angiogenesis and vessel formation is not initiated by the appearance of proangiogenic factors but by the decline of factors associated with the acute inflammatory phase. For this reason, the use of proangiogenic agents, such as VEGF for the treatment of chronic wounds has been unsuccessful in clinical trials.^69^ The equilibrium in macrophage polarization plays a pivotal role in determining the outcomes of various inflammatory conditions, encompassing cardiovascular disease, cancer, atherosclerosis, wound healing, and other pathological processes.^70^ The reversal of M1 to M2 macrophages in the further stages of inflammation is associated with the increased production of TGF-β, VEGF stimulation, and the induction of a proangiogenic program in ECs.^71^ However, in the tumor microenvironment (TME), the predisposition to M2 polarization suggests a potential contribution to tumor angiogenesis.^72^

### 3.2 Endothelial Migration in Physiological and Pathological Angiogenesis

Angiogenesis is involved in maintaining homeostasis by stimulating the formation of new blood vessels in tissue that is lacking in nutrients or oxygen. The migration of endothelial cells during the formation of blood vessels is an example of a process dependent on both collective and single-cell migration given that one EC can migrate in both ways depending on the context of migration. ECs moving individually are observed during embryonic development, whereas in adult angiogenesis EC monolayers move collectively in response to growth factors, required both to repair existing vasculature and to generate new blood vessels *in vivo*.^1,62^ New vasculature creation requires communication between tip and stalk cells (similar to the leader and follower cells in vascular repair), essential for the directed migration process in sprouting angiogenesis. The concept of tip and stalk cells involves the privilege of the tip cell to migrate in a fine-tuned feedback loop tightly controlled by VEGF. Tip cells are also polarized and scan the environment through extending filopodia that direct new blood vessels toward the chemotactic stimulus. When filopodia formation is inhibited, lamellipodia formation is sufficient to drive endothelial cell migration. Nevertheless, filopodia facilitate the migration of endothelial cells and the formation of anastomoses. The collective migration in cell cohorts and the local coordination of cell movement within sheets are mediated by genes involved in cell adhesion, such as those encoding α-catenin and VE-cadherin. Neighboring cells in sheets exhibit partial coordination in their direction of migration, while cells farther apart are relatively randomly oriented. Sprouting angiogenesis also requires the degradation of the ECM, the liberation of important growth factors sequestered within it, including bFGF, VEGF, and insulin-like growth factor, and the involvement of integrins for regulating the formation of FA at migrating cell–ECM contacts.^10^

**Figure 3:**
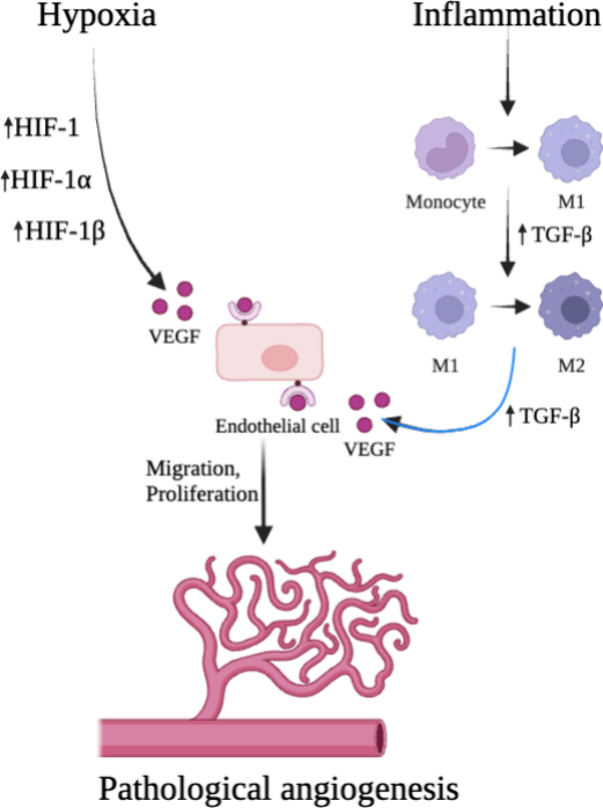
Hypoxia and inflammation in the progression of pathological angiogenesis. VEGF is the main factor contributing to the induction and progression of angiogenesis. In pathological microenvironments, such as cancer, VEGF is overproduced due to processes triggered by the lack of oxygen and immune/inflammatory responses. Accumulation of cytokines modulates the polarization of macrophages to an M2-like phenotype, which is important in promoting tumor progression and invasion. The key regulator involved in M2 activation is TGF-β, which is further released, i.e., from that macrophage and involved in the upregulation of VEGF expression. Then, overstimulated ECs begin to migrate and proliferate excessively resulting in the expansion of new blood vessels supporting tumor growth (designed with BioRender: https://biorender.com/).

Pathological microenvironments such as TME can be explained by abnormalities in the endothelial barrier structure and insufficient oxygen concentration in the affected tissue. Tumor endothelium is dysfunctional and leaky via differences in morphology, gene expression, excessive branching, and sprouting. Tumor hypoxia activates and stabilizes transcription factors, such as hypoxia-inducible factor 1 (HIF-1), a heterodimeric transcription factor that comprises an oxygen-regulated α-subunit (HIF-1α) and a constitutively expressed β-subunit (HIF-1β). This is followed by transcriptional regulation of a series of hypoxia-inducible genes, including the principal proangiogenic factor VEGF. Besides the growth factor role, VEGF is also an important factor that regulates vascular permeability. Under physiological conditions, angiogenesis associated with inflammation is required to maintain homeostasis. However, at the TME, there is an intimate connection between immune cells and the endothelium, and the inflammatory processes are dysregulated and prolonged. The resulting haphazardly shaped and leaky tumor vasculature maintains a continuous state of hypoxia, which triggers pathological angiogenesis (Figure 3).^62,73,74^

The broad spectrum of cytokine action influencing the angiogenesis process can be explained by the example of transforming growth factor-β1 (TGF-β1).^75^ TGF-β primarily regulates the activation state of endothelial cells through differential activation of two type I receptors, ALK1 and ALK5. ALK5 signaling initiates the phosphorylation of its downstream protein mediators, Smad2 and Smad3, resulting in the inhibition of angiogenesis by changes in the proliferation, migration, and organization of endothelial cells. However, endothelial cells are characterized by the high expression of ALK1, which contributes to the progression of inflammation by driving pathological angiogenesis.^76^ Moreover, the TGF-β1 signaling pathway is dysregulated in pathological states associated with vascular inflammation, which is caused by the excessive production of this cytokine by ECs. Moreover, active TGF-β1 ligands can be delivered by extracellular vesicles (EVs) derived from tumor cells and induce the transformation of the ECs. TGF-β1 is also produced and secreted by accumulating immune cells, such as monocyte-derived M2-polarized macrophages that promote the angiogenic potential of carcinoma cells *in vitro*, resulting in increased release of VEGF and bFGF from the tumor cells Therefore, the recruitment of tumor-associated macrophages (TAMs), characterized as M2-like macrophages, is a mechanism linking and driving angiogenesis and inflammation because the same cytokines can activate their accumulation and be further secreted by them to the microenvironment exhibiting effects on both processes.^72,77^

Prolonged inflammation and impaired angiogenesis have become some of the most interesting and difficult research problems often discussed in the context of chronic wounds. The most dominant proangiogenic factors in wound healing are VEGF-A produced in response to hypoxia, fibroblast growth factor -2, platelet-derived growth factor, and also members of the TGF-β family. These factors enable good control of both robust capillary growth and controlled capillary regression in normal wound-healing skin, resulting in a final vessel density that is comparable to that of normal skin. Therefore, loss of proangiogenic stimulation is necessary for capillary stabilization in the later stages of wound healing. This is performed by the activity of negative regulators of angiogenesis, such as Sprouty2, pigment epithelial origin factor, and CXCR3 ligands, such as IFN-inducible protein-10 (CXCL10). Acute inflammation in response to tissue injury leads to the recruitment of immune system cells such as macrophages producing high levels of proangiogenic factors such as VEGF, which also increases vascular permeability, making these two processes intricately connected. Excessive inflammation leads to excessive and impaired angiogenesis, and it works both ways. This implies that approaches that regulate inflammation as well as promote angiogenesis are reasonable to be effective and necessary for accelerating the healing of a wide range of chronic wounds.^78−82^

## 4 Multifaceted Effects of Biomaterials Enhance Endothelial Cell Processes in the Inflammatory Microenvironment

Interest in developing biomaterials for novel regenerative medicine applications has significantly increased in recent years, posing a major challenge. Advances in scaffolding, followed by *in vitro* culture or *in vivo* implantation, hold promise as a strategy. However, at present, this approach is hindered by the lack of specific materials and other associated difficulties.^83^ The proper migration of relevant cells is crucial for the successful integration of a medical implant with the surrounding tissue. Insufficient or excessive cell migration can lead to unfavorable effects. Endothelialization, which involves cell adhesion and migration, plays a vital role in this process, particularly in reducing thrombosis and restenosis. This aspect is of particular significance in the context of cardiovascular stents.^84^

One of the main factors that must be considered for improvement of biomaterial-based therapies is inflammation, inasmuch as it is essential for wound healing. Unfortunately, prolonged inflammation can critically affect healing processes. The body perceives the implanted materials as foreign, which leads to an inflammatory response. However, suitable biomaterial designs can modulate advantageous immune reactions that may be reflected in both anti-inflammatory and pro-regenerative ways. Therapeutic approaches targeting such responses primarily focus on biomaterial applications as carriers for distribution of anti-inflammatory bioactive compounds and pro-regenerative stem cells. Nevertheless, biomaterials are seldom used as immunomodulatory agents. Hence, it is worth noting that there are biomaterials that exhibit pro-regenerative and anti-inflammatory properties by themselves.^85−87^ However, even well selected scaffold physicochemical features along with the additive of bioactive agents retain demanding aspects.^88−90^ Furthermore, it has been established that the cellular microenvironment determines the complex signaling domain that leads to the cell phenotype. Numerous studies have proven that cells behave much more natively when cultured in three-dimensional (3D) environments.^91^ Cell migration in 3D conditions is essential in many biological developments, and its progression affects various types of processes involving inflammation, tissue regeneration, or angiogenesis. Consequently, control over cell migration is therefore crucial in both fundamental research and the development of biomaterials with improved tissue regeneration capabilities.^92−94^

Furthermore, angiogenesis is a vital process for maintaining homeostasis, including both repair and regeneration of damaged tissues. However, some pathological conditions that occur during various diseases and some inflammatory disorders lead to deregulation of this process. Additionally, angiogenesis occurs during the wound healing process through the activation of ECs in response to certain stimuli to speed tissue rebuilding.^95^ By and large, ECs maintain vascular homeostasis through a series of interactions with circulating blood components.^96^ Therefore, inflammation is essential in restoring homeostasis, and moreover, it is vital in responding to danger signals caused by damage.^97^ Although the host immune response remains the vital issue in tissue engineering, the inflammatory microenvironment makes tissue regeneration increasingly challenging. In recent years, due to the amplified amount of research, it has been confirmed that absolute evasion of inflammation is not considered advantageous for the disease’s outcome. Contrarily, it is widely acknowledged that regulated inflammatory response is required for tissue remodeling and effective regeneration. Broadly speaking of scaffolds, apart from the fact that their biocompatibility is required, they should also be targeted to capture pre-existing inflammation. Thus, there is a tendency toward transforming “immune-evasive” bioinert tissue structures into “immune-interactive”, bioactive ones, as well as enhancing their immunomodulatory capabilities to provide a perfect microenvironment for cells. Despite the lack of clinical studies, various inflammation-targeting scaffolds have shown promising *in vivo* results.

Most synthetic polymers have been found to trigger severe inflammation *in vivo*, whereas natural biomaterials elicit a significantly reduced immune response.^98,99^ Excessive proinflammatory reactions can lead to healing complications, nonfunctional fibrosis, or the degradation of transplanted biomaterials. However, this degradation, in relation to the ECM, releases matrix-related bioactive components that guide cell differentiation, proliferation, and migration. By and large, the ECM is defined as a noncellular tissue component that constitutes a physical scaffolding for cells. Apart from structural support, the ECM can deliver tissue-specific biochemical and biophysical signals. Therefore, ECM-mimicking biomaterials are considered an effective solution in the construction of scaffolding for regenerative medicine purposes.^100−102^ ECM mimics are largely used as models to study drug biodistribution in certain tissues. Such applications mostly focus on the development of 3D scaffolds and 3D bioprinting that turn out to be the most promising techniques. Countless tissue-culture prototypes are currently being developed, which enable research into the interaction of ECM biochemical and biophysical characteristics. Moreover, increasing knowledge provides better comprehension of the molecular mechanisms of cellular behaviors governed by ECM properties. Cell culture studies make use of two distinct types of scaffolds. Therefore, we can distinguish reconstituted matrices comprising biomacromolecules derived from animal tissue or artificial ECM mimics. In both cases surface coatings can be used to increase cell adherence or to create 3D scaffolds to insert cells in a more *in vivo*-like environment. The capacity to alter biophysical characteristics, such as the mechanical features or permeability of the matrix, to examine their effect on cell destiny is a key benefit of synthetic ECM. However, their inability to provide molecular signals to the cell for it to “communicate” is the root of the problem. To address this limitation, synthetic polymers can be improved through the addition of signaling biomolecules like glycans, peptides, and growth factors.^103^

The application of hydrogels as ECM mimics necessitates a thorough understanding of the cell’s native environment. The interactions between cells and their microenvironments are crucial for various processes essential for homeostasis, tissue growth, and regeneration. Among the different types of biomaterials, hydrogels are extensively used as 3D structural supports for cell culture. Remarkably, these materials can mimic many aspects of natural cellular conditions, making them valuable in tissue engineering and regenerative medicine. However, their fragility and limited mechanical strength present some challenges. Natural polymer hydrogels have high biocompatibility and biodegradability as well as minimal immunogenicity, great cytocompatibility, and controlled solubility, although they are limited by weak mechanical qualities, high production costs, and poor repeatability all at the same time. In turn, synthetic polymers are distinguished by increased mechanical strength, potential for reproduction, lower prices, and the capacity to control their composition to optimize processes such as hydrolysis or biodegradation over varying time periods. Furthermore, mechanical properties of the hydrogel determine cell self-organization, proliferation, as well as migration.^83,91,101,104^ A wide range of natural and synthetic polymers have been used to synthesize hydrogels. One such polymer is poly(ethylene glycol) (PEG), which has long been favored as a synthetic biomaterial for regenerative medicine applications. PEG is a nonionic water-soluble polymer known for its biocompatibility. Some studies have combined PEG with other polymers for the targeted delivery of angiogenic substances. Additionally, certain PEG derivatives, like poly(ethylene glycol) dimethacrylate (PEGDMA), have garnered interest as intriguing targets.^95,105^ Poly(ethylene glycol)-coupled polymers enhance angiogenesis while also transporting medications or bioactive molecules to the site of damage, thus reducing the inflammatory response. Since these polymers may pass both the blood–spinal cord and blood–brain barriers, they are commonly utilized as medication carriers. Additionally, PEG inhibits cell apoptosis by keeping cell membranes unimpaired. With 8-arm poly(ethylene glycol) (PEG8a) macromers in conjunction with endothelial cells, endothelial network development increased significantly.^106−109^

It is worth noting that natural biomaterials, which structurally resemble native tissues, possess the ability to reduce foreign body effects and trigger initiation of the remodeling reaction. Therefore, protein-based hydrogels are among the most commonly used scaffolds in tissue engineering. These hydrogels are developed using proteins obtained from the extracellular matrix (ECM) or other biological sources. They are attractive solutions because proteins naturally promote cell adhesion and proliferation.^83^ Collagen, the most prevalent protein in the ECM, especially type I collagen, is widely utilized as a natural scaffold in research. Within hydrogels, proangiogenic substances and endothelial cells (ECs) can be incorporated to promote angiogenesis. Collagen hydrogels facilitate the construction of 3D microcapillary networks by ECs and perivascular cells. However, it is essential to mention that this type of scaffold induces only a mild inflammatory response. Additionally, hydrolysis of collagen leads to gelatin formation, and when combined with hydrogels, it enables cell adhesion and mobilization of ECs and promotes angiogenic healing through the release of various molecules.^110^ Modified scaffolds, such as methacrylate gelatin (GelMA), promote endothelial growth by delivering VEGF and offer an alternative for 3D printing of tube-like structures.^14^ Fibrin exhibits similar properties and plays a meaningful role in processes such as wound healing, maintaining homeostasis, and modulating the inflammatory response. Fibrin hydrogels are also excellent choices for creating 3D blood vessel capillaries as they can accommodate both endothelial and mesenchymal cells. The ability of hydrogels to release growth factors further contributes to their proangiogenic properties. Hydrogels derived from natural sources and enhanced with specific chemical compositions, such as hyaluronic acid (HA), can also serve as the foundation of a proangiogenic system. HA-based hydrogels are characterized by superior biodegradability, high viscoelasticity, hydrophilicity, and biocompatibility. Moreover, HA reduces platelet adhesion and aggregation while stimulating angiogenesis, making it suitable for vascular applications. The production of hydrogels based on combinations of synthetic and natural polymers offers the potential to optimize their characteristics and create appropriate scaffolds. Thus, the development of new, stronger, and more stable hydrogels with improved biocompatibility remains a crucial goal.^110^

The pro- and antiangiogenic potential of nanomaterials can be easily regulated through surface modifications, self-assembly, and minor alterations to the synthesis process. Moreover, certain nanomaterials can condition the course of angiogenesis by releasing therapeutic ions. Acting as carriers, nanomaterials efficiently distribute proangiogenic factors. Nanoparticles (NPs) offer excellent control over scaffold features, such as mechanical strength and the controlled release of various bioactive agents.^95,111,112^ Interestingly, it was found that nanofiber scaffolds filled with fibroblast growth factor (FGF) or VEGF increase angiogenesis and reduce thrombosis when compared to those without them. Moreover, such materials can mimic the nanotopological structure of blood vessel ECM, which makes them beneficial for migration, proliferation, and adhesion of endothelial cells. Regarding the ECM of vascular tissue, which is nanostructured, nanomaterials are extremely valuable as biomimetic vascular tissue scaffolds. Owing to their structure and unique properties, they are increasingly used in biomedical applications. Electrospinning may be widely utilized to create nanofibrous scaffolds. In preliminary studies, electrospinning appears to be a potential approach for vascular grafts. Furthermore, the advancement of this process, as well as the creation of electrospun nanofibers to suit or enable various applications, has made remarkable progress. Moreover, nanofibers may be manufactured from a wide range of materials.^113^ Broadly speaking, this electrohydrodynamic technique includes electrification of a liquid droplet to generate a jet. Afterward it is stretched and elongated, which results in formation of fiber(s). This technique makes stress sustained by embedded nanofibers, which leads to reinforcement of matrix elasticity.^114^ It was revealed that using nanofiber scaffolds improves the adhesion and proliferation of ECs, while also having a progressive impact on the angiogenesis process. However, some studies have shown that such properties of ECs increase with scaffold stiffness. The harder the substrate is, the farther ECs migrate, but also they deposit on fibronectin fibers in a more linear and aligned manner. Additionally, apart from the influence on cell differentiation and motility, this characteristic also controls the absorption of the nanoparticles. Metal-based nanoparticles with use of gold and cerium oxide have proangiogenic effects, which make them beneficial for wound healing. Likewise, functional peptide-coated gold nanoparticles support angiogenesis along with endothelial cell capillary formation. Electrospun nanofibers considerably improve endothelialization, which is an effective method to prevent thrombosis. Electrospinning together with hot embossing was also used to make three-dimensional poly(l-lactic acid) nanofibrous scaffolds, which affected the endothelial cells, improving their focal adhesion growth and proliferation.^113,115^ One study has demonstrated that nanofibrillar collagen scaffolds effectively modulate the endothelial inflammatory response and guide the organization of endothelial cells, encompassing both cytoskeletal and nuclear components of ECs.^96^ Furthermore, these materials have been shown to enhance cell survival after implantation, in both ischemic and normal tissues. Generally, unmodified scaffolds exhibit a limited capacity for tissue regeneration and disease treatment. As a result, there is growing interest in liposome scaffold composite systems, which offer several advantages. These scaffolds leverage the biocompatibility of liposomes, along with the durability of the scaffolds. Liposomes themselves boast benefits such as low toxicity, nonimmunogenicity, and biodegradability. Initially considered as cell membrane models, they are now primarily utilized as carriers for medications. Phospholipid vesicles, forming lipid bilayers, are the most common form of lipid nanoparticles capable of delivering various chemicals, medicines, and genes. Furthermore, liposomes possess the ability to modulate medication release and reduce pharmacological adverse effects.^116^ This capacity is especially crucial for maintaining effective concentrations of certain regulators, such as FGF, at the wound site during the wound-treatment process. FGF, in particular, supports the development of new blood vessels, aids in repairing damaged ECs, and promotes their migration.^95^ In the future, the combination of scaffolds and liposomes may lead to the creation of self-assembled drug carrier systems, offering promising prospects for tissue regeneration and disease treatment.^116,117^

### 4.1 The Influence of Physicochemical Properties of Surface in the Context of Endothelial Cell Behavior

**Figure 4:**
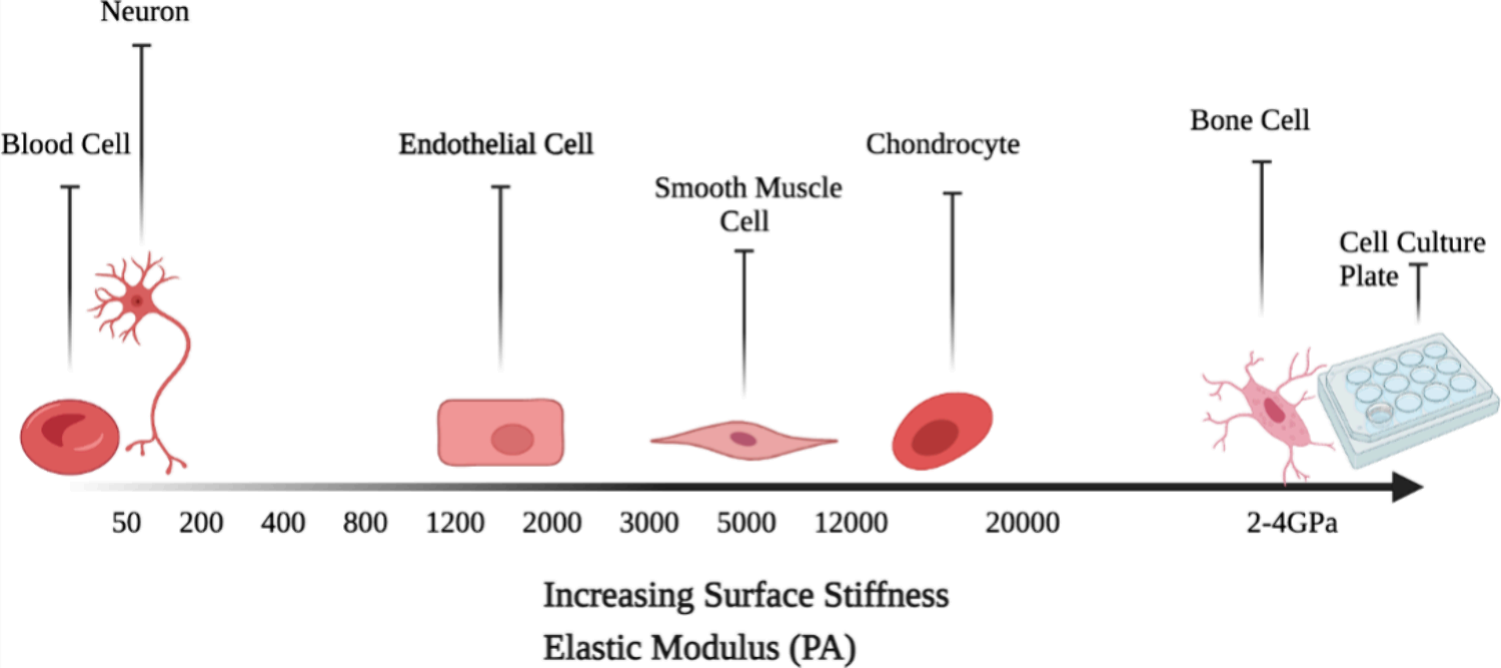
Impact of substrate stiffness on cellular phenotype. Substrate stiffness has been found to influence cell stress fibers as well as focal adhesions in different cell categories. Cell function is constantly modulated by the mechanical surroundings through cytoskeletal alterations and actomyosin retractility (designed with BioRender: https://biorender.com/).

The ECM stiffness and nanotopographical properties impact many developmental, physiological, and pathological processes. Therefore, these biophysical signals have been used to influence practically every aspect of cell activity, from cell adhesion and spreading to differentiation and proliferation. Delineating the biophysical control of cell behavior is crucial for the rational design of novel biomaterials and implants, as well as medical devices. It is a key aspect to clarify the connection between extracellular regulation of cell behavior and the biophysical cues. It has been reported that the stiffness of the ECM is determined by its composition and the presence of interstitial fluids.^118^ Furthermore, the cell phenotype and function are regulated by biophysical signals in conjunction with spatiotemporally organized biochemical and biomechanical cues. Cells frequently demonstrate better cell adhesion, further cell spreading with defined actin organization, increased cellular contractility, slowed migratory speed, and enhanced proliferation as substrate stiffness increases (Figure 4).^118,119^ The majority of biomaterials used for cardiovascular device surfaces are not biocompatible with the establishment of an endothelial layer. Plentiful research has primarily aimed to change these surfaces by physical, chemical, and biological mechanisms in order to facilitate early EC adhesion, migration, and proliferation to ultimately form an endothelial layer on the surfaces. Surface modification involves inhibiting protein adsorption, which prevents platelet attachment to device surfaces and may enhance EC adhesion. Surface alteration by texturing, if applicable, can yield some promising results in this area. Surface changes by chemical/biological techniques may play an important role in the easy endothelialization of cardiovascular devices and the inhibition of smooth muscle cell proliferation, as well. Cellular engineering of endothelial cells can enhance the beneficial effects acquired by surface engineering.^120,121^

Material features govern their interactions with cells and tissues and have a significant impact on cell migration, adhesion, differentiation, and proliferation. Furthermore, several materials with proper cell responses have been explored in order to develop implantable devices or other therapeutic implants in the medical industry. Surface modification of biomaterials to enable fast endothelialization on implants has been one of the core subjects for cardiovascular applications. One study proposed a LAA (left atrial appendage) occlusion device with a well-designed 3D architecture and a nanoscale 2D coating for interventional therapy. The efficacy of the nanocoated LAA occluder was confirmed in animal tests and a patient case, both of which demonstrated successful implantation, rapid sealing, and long-term device safety. This research shows that nanocoating improves cell migration on modified 2D surfaces. It has been demonstrated that HUVECs (human umbilical vein endothelial cells) migrate much faster on TiN-coated surfaces than on bare NiTi alloy. Both *in vivo* and *in vitro* studies have demonstrated that medical devices with nanocoatings endothelialize faster than those without surface modification. An important conclusion that was drawn during the research concerned the fact that a significant increase in cell adhesion on a material surface does not necessarily promote cell migration. The fastest migration occurred under moderate cell adhesion, which leads to the statement that cell adhesion and migration may be associated non-monotonically.^122^

While several ways to improve cell adhesion and so on have been proposed, inherently little is still known about the strategy to increase cell migration on a nanoscale alteration. Another study investigated the effect of arginine-glycine-aspartate (RGD) peptide nanospacing on cell migration and its relationship to cell adhesion. RGD nanopatterns with varying nanospacings (31–125 nm) on a nonfouling backdrop of poly(ethylene glycol) were created for this purpose. Afterward, HUVECs were used to study cell behaviors on the nanopatterned surfaces. The cells attached effectively to surfaces with RGD nanospacing less than 70 nm and exhibited a monotonic decline in adherence with an increasing RGD nanospacing. Furthermore, the highest migration velocity was around 90 nm for nanospacing, with sluggish migration occurring in both vastly smaller and larger RGD nanospacings. Considering that overly weak cell adhesion may not support the formation of a new leading edge and extremely strong adherence may prevent the retraction of a trailing edge, it was anticipated that cell migration would alter nonmonotonically with RGD nanospacing. As a result, modifying a biomaterial with reduced cell adhesion may result in improved cell migration. Moreover, a suitable nanoscale alteration might considerably improve cell migration, whereas another nanoscale adjustment may have the opposite effect. These findings might be highly useful in directing the design of biomaterials for encouraging tissue regeneration with increased cell migration or limiting tumor metastasis with reduced cell migration.^123^

Various surface modification approaches have been suggested to promote ECs adhesion on the normally nonadhesive polymeric biomaterials utilized for synthetic vascular grafts. However, the obstacle is the rate and quality of endothelialization, which is dependent on EC interactions with such implants and determines cell adhesion and migration. Even though the interaction between these two mechanisms is established, how surface modification exploiting covalently bound adhesive peptides affects cell movement remains obscure. Therefore, scientists decided to conduct a study to identify the impact of surface changes on endothelial cell migration. Research aimed at utilizing amino phase glasses modified with the covalent immobilization of Gly–Arg–Gly–Glu–Tyr (GRGEY), Arg–Gly–Asp–Ser (RGDS), and Tyr–Ile–Gly–Ser–Arg–Gly (YIGSRG) adhesive peptides settled on a biomaterial surface. To follow and assess the movement of a large number of cells, this work used an upgraded migration experiment that integrated spatially enhanced video microscopy with a digital time-lapse recording. The digital pictures were then examined in order to reconstruct cell trajectories and compute locomotory metrics, such as random motility coefficient, migration speed, and persistence duration. By this approach, the interactions of ECs along with substrates modified with covalently attached adhesive peptides were studied. The persistence of cell movement and consequently the random motility coefficient were dramatically enhanced. Specifically, the persistence time for ECs migration on the YIGSRG-modified glass was meaningfully higher. These findings imply that immobilizing cell adhesion peptides on the surface of implantable biomaterials may increase endothelialization rates.^124^

EC migration and proliferation are involved in the wound healing process following vascular damage. The enhanced generation of nitric oxide (NO) throughout this process is valuable due to its favorable effects on vasodilation and platelet aggregation prevention. It has been demonstrated that the NO regulates EC development, migration, and angiogenesis. Endothelial nitric oxide synthase (eNOS) is constitutively expressed in ECs and constitutes a significant component in vascular tissue protection. Broadly speaking, the phosphoinositide 3-kinase (PI3K) signaling pathway is a critical regulator of EC proliferation and survival. Furthermore, a serine/threonine protein kinase called Akt is attracted to the cell membrane through its interaction with PI3K. Akt then phosphorylates and activates eNOS, resulting in NO production and further EC development and migration. Since eNOS and PI3K/Akt have tight interactions and can affect EC activities, it was expected that activating eNOS would drive ECs grown on polyurethane (PU) to migrate via the PI3K/Akt signaling pathway. Therefore, the goal of one research project was to investigate the processes of eNOS-induced EC migration and proliferation on biomaterials with various surface morphologies, utilizing a unique series of PU nanocomposites as a model system. The nanocomposites made of PU combined with several low concentrations (17.4–174 ppm) of gold nanoparticles (PU-Au) were utilized as a model system to describe the mechanisms that affect migration of ECs seeded on biomaterial surfaces. A real-time imaging system was used to determine the migration rate of the ECs on PU-Au nanocomposites. The migration rate of ECs was reported to be the highest on the nanocomposite containing 43.5 ppm of gold (“PU-Au43.5ppm”). Increased levels of endothelial nitric oxide synthase (eNOS) and phosphorylated Akt (p-Akt) produced by ECs grown on PU-Au were related to the high EC migration rate.^125^

One study focused on the Cys-Ala-Gly (CAG) peptide density gradient has demonstrated that it does not interact with blood cells, making it suitable for cardiovascular applications. Moreover, CAG-modified biomaterials have been shown to dramatically improve EC proliferation and adhesion over smooth muscle cells (SMCs), resulting in rapid endothelialization both *in vitro* and *in vivo*. However, the mechanism of the CAG peptide–cell interaction remains unknown and, its impact on the migration of ECs is yet to be determined. Therefore, another approach for producing an EC-affinitive peptide gradient on homogeneous resistive polymer brushes was established and presented, with the goal of attaining selective EC adherence over SMCs, as well as directed and quicker migration of ECs driven by gradient cues. In this study, the CAG peptide sequence was selected from specially enriched tripeptides in collagen type IV, which is a significant component of the ECM basement membrane of blood vessels that divides ECs and SMCs in vascular tissues. SMC adherence and dissemination were successfully reduced at all sites. At 10 mm of gradient in coculture conditions, adherent ECs outnumber SMCs by around 6-fold. Owing to the gradient signal and suitable contact with the substrate, ECs migrated the fastest with a directivity of 86.7% at the center of the gradient, resulting in the largest net displacement as well. The CAG gradient enabled ECs to migrate in a directed manner while retaining SMC resistance, making it more appropriate for fast endothelialization.^126^

Another study sought to create a biodegradable star-shaped copolymer, poly(lactide-*co*-3(*S*)-methyl-morpholine-2,5-dione)_6_ (Star-(PLMD)_6_) through ring-opening polymerization (ROP). Following that, a gene carrier Star-PLMD-*g*-PEI-*g*-PEG-CREDVW was created by grafting polyethylenimine (PEI), poly(ethylene glycol) (PEG), and the targeting peptide REDV onto Star-(PLMD)_6_. Throughout the electrostatic interaction, this gene carrier was able to produce stable micelles and condense pEGFP-ZNF580. The REDV peptide was preferentially localized on the surface of the micelles, endowing them with EC-targeting properties. These micelles had a high capacity for pDNA condensation and were not harmful to the ECs. The transfection impact and level of associated protein expression in ECs demonstrated that the targeting complex group was much greater than the control and nontargeting groups. The resultant complexes were biocompatible and effective in gene delivery. These compounds demonstrated great EC selectivity and high transfection efficiency. Furthermore, the migration and proliferation of cells treated with targeting complexes were significantly enhanced, which demonstrates a high potential for targeting complexes on endothelialization.^127^

Cardiovascular disease is the top cause of mortality worldwide. Stent implantation restores blood flow perfusion by extending the vascular wall, which leads to reconstruction of stenotic arteries. However, stent settlement invariably results in endothelial denudation, which increases in-stent restenosis (ISR), as well as late thrombosis. Rapid re-endothelialization is a key treatment target for avoiding ISR and thrombosis. Therefore, the main factor directing this mechanism is the migration of vascular endothelial cells (VECs) near the injured intima. Furthermore, *in vitro* models were created to replicate various endothelial denudation scales (2 mm/5 mm/10 mm), as well as stent deployment depths (flat/groove/bulge) to assess the combined contribution of VEC migration and adhesion to re-endothelialization under flow and the effect of stent. Findings of this study revealed that in 2 mm flat/groove/bulge models, VEC migration and adhesion together completed about 27%, 16%, and 12% of endothelium recovery, correspondingly, while migration accounted for approximately 21%, 15%, and 7%. Endothelial repair was revealed to be mostly dependent on the migration of neighboring VECs at a 2 mm damage scale; however the quantity of circulating VEC adhesion increased and significantly contributed to endothelial repair as the injury scale grew, indicating an injury scale dependence.^128^

The damage of ECs during stent implantation might result in serious after-effects, such as restenosis. Due to many studies, surface biocompatibility is constantly being improved to speed stent endothelialization in order to prevent restenosis. Therefore, researchers aimed at anodization on Ni–Ti, which establishes a ready and effective surface modification process for improving the biocompatibility of Ni–Ti stent surfaces by increasing hydrophilicity, causing an increase in EC activity. To investigate the flow influence on the EC behavior, a parallel plate flow chamber was developed to provide a constant wall shear stress (WSS). The hydrophilicity of the Ni–Ti stent strut surface was improved by an anodized TiO_2_ coating. The EC adhesion and morphology on the anodized stent strut were examined both with and without flow. ECs under the static condition (without flow) showed superior concentration on the surface of the anodized Ni–Ti stent strut contrasted with the control, whereas in the flow condition, the improvement of the EC density was reduced. The ECs revealed an extended and lean spindle-shaped morphology under the flow condition. Anodization may improve EC migration onto the strut surface and hence speed the Ni–Ti stent endothelialization process by increasing the surface hydrophilicity. Furthermore, surface hydrophilicity expands less under flow circumstances than under static ones.^129^

Polymeric biomaterials used for regenerative medicine must be biocompatible, bioactive, and bioinert. Nevertheless, cells do not interact with surfaces unless the surfaces are coated with adhesive proteins or peptides. Industries routinely modify the surface characteristics of bulk polymer materials to achieve biocompatibility. Plasma surface modification is an influential technique for modifying material characteristics and controlling cell activity on a surface. The efficiency of a plasma polymerized 4,7,10-tridecanediamine (ppTTDDA) film coated on a polystyrene (PS) Petri dish, which is a biocompatible surface containing carbon- and oxygen-based chemical species, was studied. Furthermore, the effects of ppTTDDA on bovine aortic endothelial cell (BAEC) migration were also examined. A cell movement analysis was carried out, and cell migration was measured for up to 12 h and expressed as a percentage of the number of cells that moved after scraping divided by the cells present before making the wound. Observations revealed that ppTTDDA-coated PS may interact with cells directly, regardless of the cell adhesion molecules. The significant concentration of carboxyl groups on the surface of the ppTTDDA film was related to the improved cell affinity. The carboxyl surface showed a remarkable capacity to enhance BAEC culture. Plasma surface modification approaches improve biocompatibility while also providing a surface environment for cell growth (Table 1).^130^

**Table 1:** Overview of Various Biomaterials Utilized in Regenerative Medicine Applications, Including Their Diverse Applications, Properties, and Examples, Showcasing Their Significance in Tissue Engineering and Regenerative Therapies

biomaterial	application/function	key properties	representative examples
synthetic polymers	scaffolding, drug delivery	tailored mechanical features, controllable composition	poly(ethylene glycol) (PEG), PEG derivatives (PEGDMA)
natural polymers	scaffolding, hydrogels	high biocompatibility, biodegradability, minimal immunogenicity	collagen, fibrin, hyaluronic acid (HA)
nanomaterials	pro/anti-angiogenic	surface modifications, therapeutic ion release	gold and cerium oxide nanoparticles, functional peptide-coated gold nanoparticles
hydrogels	3D structural support	mimic natural cellular conditions, valuable in tissue engineering	collagen-based hydrogels, fibrin hydrogels, HA-based hydrogels
liposomes	drug delivery, cell interaction	biocompatible, nonimmunogenic, biodegradable	liposome-scaffold composite systems
polyurethane (PU)	EC migration, gene delivery	various surface morphologies, impact EC behavior	PU nanocomposites, PU–Au nanocomposites
nanofibers	endothelialization, cell adhesion	mimic blood vessel nanotopological structure	electrospun nanofibrous scaffolds, metal-based nanoparticles
peptides	EC affinity, selective adherence	promote EC proliferation and adhesion	Cys-Ala-Gly (CAG) peptide density gradient, REDV peptide
ECM mimics	tissue regeneration	mimic extracellular matrix, support cell differentiation and migration	collagen, fibrin, hyaluronic acid-based hydrogels
surface modification	endothelialization	modifies surfaces for fast endothelialization	anodization on Ni–Ti stent surfaces, plasma surface modification (ppTTDDA film)

## 5 Conclusion

Endothelial cell migration plays a fundamental role in numerous physiological and pathological processes, and it is influenced by several factors including proinflammatory agents and properties of the cellular substrate. Immunoregulatory cytokines and growth factors, which favor inflammation, can either promote or inhibit cell movement, depending on the specific stage of inflammation. This aspect should be considered when targeting the activity of specific molecules. Crucially, sustained angiogenesis and inflammation share significant signaling pathways and molecules. Inflammatory mediators such as TGF-β may lead to abnormal cell movement, resulting in impaired migratory properties that underlie pathological neovascularization. This phenomenon can be related to tumor metastasis, plaque growth, and instability or the failure of chronic wounds to heal. A key goal is to understand the subtle differences in cell signaling that affect the migration process under homeostatic and inflammatory conditions, enabling the development of strategies that preserve the physiological function of the endothelium. Achieving a molecular balance is challenging, but harnessing the properties of biomaterials and creating well-designed scaffold structures may be crucial for promoting cell proliferation, migration, and functional angiogenesis. Despite the wide array of available biomaterials, researchers continuously seek new solutions to find the perfect scaffold that mimics native tissue tailored for specific purposes. Advancing our knowledge of cell–material interactions, particularly understanding the mechanisms of cell behavior in relation to different scaffold types, will undoubtedly lead to improved treatment procedures. These advancements aim to modulate immune responses toward anti-inflammatory and prohealing characteristics.

## Abbreviations

CCR2: CC chemokine receptor 2
CCL2: CC chemokine ligand 2
CCR5: CC chemokine receptor 5
TLC: thin layer chromatography
